# Development of a Clinical and Laboratory-Based Predictive Nomogram Model for Unfavorable Functional Outcomes Among Patients Who Undergo Interventions for Aneurysmal Subarachnoid Hemorrhage

**DOI:** 10.3390/jcm14051443

**Published:** 2025-02-21

**Authors:** Zhongxiao Wang, Ting Liu, Yue An, An Xu, Kangxu An, Ying Zhang, Jian Liu, Kun Wang, Wenqiang Li, Guangshuo Li, Xingquan Zhao, Weixin Si, Yisen Zhang, Xinjian Yang

**Affiliations:** 1 Department of Interventional Neuroradiology, Beijing Neurosurgical Institute, Capital Medical University, Beijing 100070, China; 2 Department of Neurosurgery, Beijing Tiantan Hospital, Capital Medical University, Beijing 100070, China; 3 Department of Neurology, Beijing Tiantan Hospital, Capital Medical University, Beijing 100070, China; 4 Shenzhen Institute of Advanced Technology, Chinese Academy of Sciences, No. 1068, Xueyuan Avenue, Shenzhen 518055, China

**Keywords:** aneurysmal subarachnoid hemorrhage, outcome, perioperative changes in laboratory indicators, nomogram, prognostic model

## Abstract

**Objective:** This study elucidates the prognostic significance of perioperative changes in laboratory indicators for aneurysmal SAH and develops a nomogram model for outcome prediction. **Methods:** Aneurysmal SAH patients who received clipping or coiling at our institution between January 2016 and December 2022 were included. All patients were randomly assigned to derivation and validation cohorts. Independent predictors of unfavorable outcomes were identified by multivariate analyses. Three models were conducted to evaluate whether perioperative laboratory changes improve prediction performance. A nomogram including all independent predictors was developed in the derivation cohort and verified in both cohorts. **Results:** Diabetes mellitus [OR (95% CI) = 2.84 (1.44–5.59)], WFNS grade 3–5 [OR: (95% CI), 9.17 (5.49–15.33)], clipping [OR (95% CI) = 1.71 (1.03–2.85)], perioperative changes in white blood cell count [OR (95% CI) = 2.15 (1.17–3.96)], and concentrations of ALT [OR (95% CI) = 1.41 (1.04–1.91)], sodium [OR (95% CI) = 5.40 (3.01–9.71)], and glucose [OR (95% CI) = 2.18 (1.05–4.53)] were independent predictors of an unfavorable outcome. The predictive nomogram incorporated the aforementioned predictors and performed well in the derivation cohort (AUC, 0.839; 95% CI: 0.810–0.866) and the validation cohort (AUC, 0.797; 95% CI: 0.734–0.850). **Conclusions:** Perioperative changes in laboratory indicators can be predictors of unfavorable outcomes in aneurysmal SAH patients. The nomogram based on clinical and laboratory risk factors can be used as a convenient tool to facilitate individualized decision making.

## 1. Introduction

Aneurysmal subarachnoid hemorrhage (aSAH) is a severe form of stroke, with a mortality rate of up to 35%. Among survivors, approximately one-third suffer from long-term severe disability and functional dependence [1,2]. The prognosis of aSAH patients is influenced not only by the intrinsic damage caused by the hemorrhage, but also significantly by treatment modalities [3,4,5].

Several prognostic tools, such as the Hunt–Hess and World Federation of Neurological Surgeons (WFNS) grading systems, are available to assess aSAH prognosis at admission and aid in decision making [6]. However, these scoring systems primarily depend on clinicians’ subjective judgment, lacking objective, quantifiable indicators. Furthermore, they fail to consider the potential impact of interventions on patient outcomes, limiting their utility in accurate prognostication.

Biomarkers that reflect physiological status have significant clinical predictive value. Previous studies have identified serum sodium, glucose, and white blood cell count as independent predictors of poor prognosis in aSAH patients [7,8,9,10,11,12]. Additionally, perioperative changes in laboratory indicators can provide insights into the physiological effects of therapeutic interventions [13,14]. Therefore, this study aims to explore the association between perioperative changes in laboratory indicators and functional outcomes. Based on these findings, we developed and validated a novel nomogram model to enhance aSAH patient management.

## 2. Materials and Methods

### 2.1. Ethics Approval

This study was conducted at the Tiantan Hospital, Capital Medical University, from January 2016 to December 2022. The approval number given by the ethical board was KY 2023-261-01. Informed consent for the clinical data was obtained from either individual participants or their authorized relatives.

### 2.2. Patients

**Figure 1:**
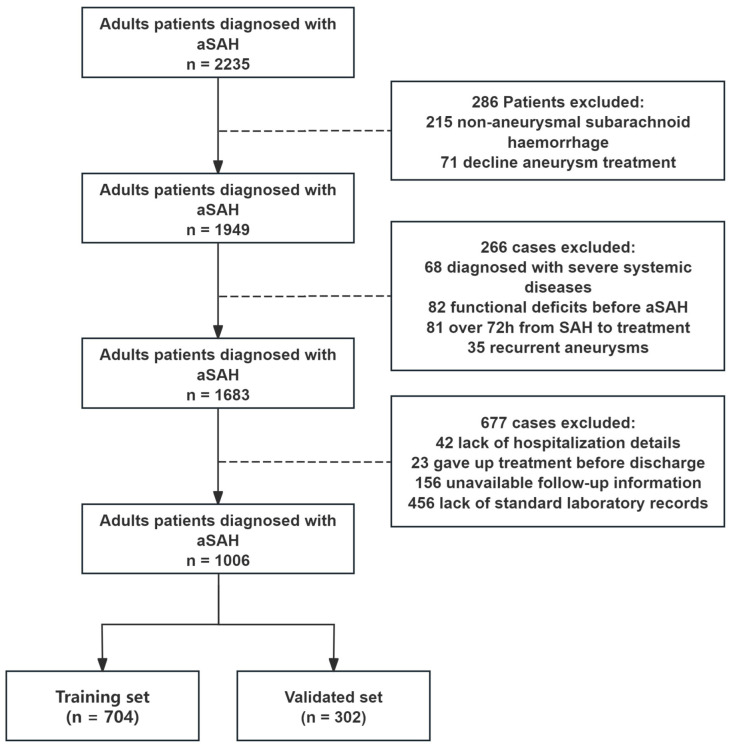
Baseline comparison between the training set and the validation set.

Patients were included based on the following criteria: (1) subarachnoid hemorrhage confirmed by computed tomography (CT), (2) intracranial aneurysm detected through CT angiography (CTA) or digital subtraction angiography (DSA) and associated with bleeding, and (3) clipping or embolization procedures conducted after admission. Exclusion criteria encompassed (1) patients who left the hospital before completing the treatment, (2) severe systemic diseases such as major liver, kidney, or heart dysfunction and hematological disorders, and (3) insufficient clinical, imaging, or laboratory data or follow-up information. Patients were randomly assigned to derivation (70% of data) and validation cohorts (30% of data) for nomogram development and validation, respectively. A flowchart is shown in Figure 1.

### 2.3. Data Collection

Patient information was obtained from electronic medical records and included the following: (1) demographic information (age, sex, smoking, and alcohol); (2) disease history (hypertension, hyperlipidemia, and diabetes mellitus); (3) neurological status (WFNS grade) [6]; (4) radiological characteristics (modified Fisher grade and aneurysm location) [15]; (5) treatment modalities (clipping and coiling); (6) laboratory indicators focusing on white blood cell (WBC) count, the neutrophil–lymphocyte ratio (NLR), red blood cell (RBC) count, hemoglobin (Hb), platelet (PLT) count, D-dimer, fibrin degradation products (FDP), potassium, sodium, glucose, estimated glomerular filtration rate (eGFR), alanine aminotransferase (ALT), and aspartate aminotransferase (AST). The NLR was calculated by dividing the neutrophil count by the lymphocyte count. As a validated inflammatory marker, NLR provides a more efficient predictor of outcomes. We used NLR in place of separate neutrophil and lymphocyte counts to reduce multicollinearity and streamline the model. Variables were assessed within 24 h of both admission and treatment. For each laboratory indicator, perioperative changes are reported as a ratio of post-treatment to pre-treatment.

### 2.4. Outcome Assessment

The primary measure was the functional status at six months post-discharge, determined by the six-point modified Rankin scale (mRS). An mRS score of 0 to 2 was considered a favorable outcome, while a score of 3 to 6 was deemed unfavorable. Functional outcomes were assessed using data obtained during outpatient clinic visits and/or telephone interviews [16,17].

### 2.5. Development and Validation of the Predictive Model

Variables showing *p* < 0.10 in the univariate analysis were included in the multivariate analysis, with those having *p* < 0.05 identified as independent risk factors. These factors were categorized into three groups: neurological status at admission, medical history and treatment modality, and perioperative laboratory indicator changes. To assess the impact of perioperative indicators on predictive efficacy, three models were developed: Model 1 included the neurological status at admission, Model 2 added medical history and treatment modality, and Model 3 further incorporated perioperative laboratory indicator changes. The complete predictive model—Model 3—is visualized as a nomogram. Model performance was assessed by examining its discrimination, calibration, and clinical effectiveness. Discrimination was assessed by the area under the receiver operating characteristic (ROC) curve, with the DeLong’s test used for performance comparison. Calibration curves were plotted to assess agreement between the predicted outcomes and the actual observations, where alignment near the 45-degree line indicates an ideal calibration. Model fit was assessed using the Hosmer–Lemeshow test (*p* > 0.05 indicating good fit). Decision curve analysis (DCA) evaluated clinical effectiveness by calculating net benefits across various threshold probabilities. Internal validation was conducted using the bootstrapping method with 1000 resamples, from which the mean AUC and its range were derived. Additionally, the validation cohort was used to assess model performance, focusing on discrimination, calibration, and clinical effectiveness.

### 2.6. Statistical Analysis

Statistical analyses were conducted using the SPSS software, version 27 (IBM Corp., Armonk, NY, USA), and the R software (version 4.1.2, The R Foundation for Statistical Computing). Normality of continuous data was assessed using the Kolmogorov–Smirnov (KS) test. Normal variables are reported as means with the standard deviation and were compared using the Student’s *t*-test. Non-normal continuous variables are reported as medians with the interquartile range and were compared using the Mann–Whitney U test. Categorical variables are reported as numbers with percentage and were compared using the χ^2^ test.

## 3. Results

### 3.1. Study Population

During the study period, 2235 consecutive patients were diagnosed with aneurysmal subarachnoid hemorrhage in our center. Among the 1006 patients who met the inclusion criteria, 153 (15.2%) had an unfavorable outcome, while 853 (84.8%) achieved favorable outcomes. A total of 704 patients (including 101 with unfavorable outcomes) were randomly assigned to the derivation cohort, and the remaining 302 patients (including 52 with unfavorable outcomes) were allocated to the validation cohort. In the derivation cohort, 59.94% (422) of the patients were female, with a mean age of 56.25 ± 11.21 years. An unfavorable functional outcome at 6 months was observed in 14.35% of the patients. Patient baseline characteristics are depicted in the Supplementary Table S1.

### 3.2. Comparison of Baseline Characteristics and Laboratory Indicators in the Derivation Cohort

Univariate analysis identified risk factors significantly associated with unfavorable outcomes in patients with aSAH. The clinical parameters of the derivation cohort are shown in Table 1. Age, hypertension, diabetes mellitus, WFNS grade, modified Fisher grade, and treatment modalities were common among patients with unfavorable outcomes (*p* < 0.05). Perioperative changes in laboratory indicators according to the group are shown in Table 2. Significant differences in perioperative changes were observed between the favorable and unfavorable outcome groups in FDP (1.12 and 1.20, respectively; *p* = 0.010), ALT (0.81 and 0.90, respectively; *p* = 0.002), AST (0.83 and 1.01, respectively; *p* = 0.009), sodium (1.01 and 1.03, respectively; *p* < 0.001), and potassium (0.96 and 1.00, respectively; *p* = 0.016), eGFR (1.02 and 0.99, respectively; *p* = 0.001), WBC (0.93 and 1.00, respectively; *p* = 0.011), and NLR (0.76 and 0.98, respectively; *p* = 0.012).

### 3.3. Prognostic Laboratory Indicators of Unfavorable Outcomes

**Figure 2:**
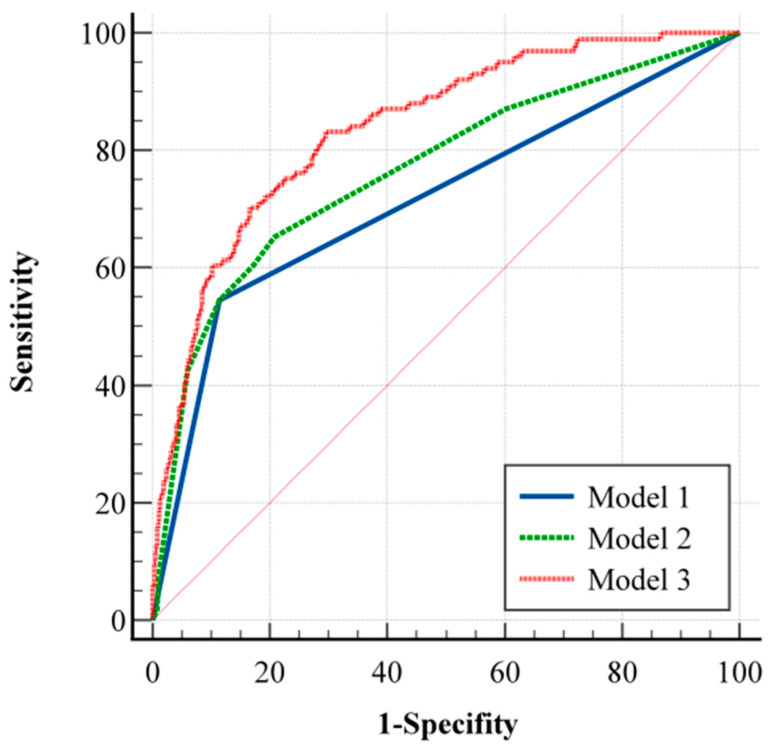
Receiver operating characteristic curve analysis comparing Model 1 (AUC = 0.715), Model 2 (AUC = 0.767), and Model 3 (AUC = 0.839) for predicting unfavorable outcomes at 6 months.

In the multivariate logistic analysis in which only variables with *p* < 0.10 were included, based on the results of the univariate analysis, diabetes mellitus (OR (95% CI) = 2.84 (1.44–5.59); *p* = 0.002), WFNS grade (OR (95% CI) = 9.17 (5.49–15.33); *p* < 0.001), treatment modalities (OR (95% CI) = 1.71 (1.03–2.85); *p* = 0.037), and perioperative changes in ALT (OR (95% CI) = 1.41 (1.04–1.91); *p* = 0.028), sodium (OR (95% CI) = 5.40 (3.01–9.71); *p* < 0.001), glucose (OR (95% CI) = 2.18 (1.05–4.53); *p* = 0.037), and WBC (OR (95% CI) = 2.15 (1.17–3.96); *p* = 0.014) were identified as independent prognostic factors (Table 3). Three predictive models for unfavorable outcomes were constructed to judge the prognostic value of perioperative changes in laboratory indicators. Model 1 included only the WFNS grade, Model 2 added diabetes mellitus and treatment modalities, and Model 3 incorporated perioperative changes in ALT, sodium, glucose, and WBC alongside Model 2’s variables. As shown in Figure 2, Model 3 achieved the highest predictive accuracy (AUC = 0.839; 95% CI, 0.810–0.866), followed by Model 2 (AUC = 0.767; 95% CI, 0.734–0.798) and Model 1 (AUC = 0.715; 95% CI, 0.680–0.748). Significant differences in AUC were observed among the models, indicating that incorporating perioperative laboratory marker changes into traditional models substantially enhances their predictive performance.

### 3.4. Nomogram Construction and Validation

**Figure 3:**
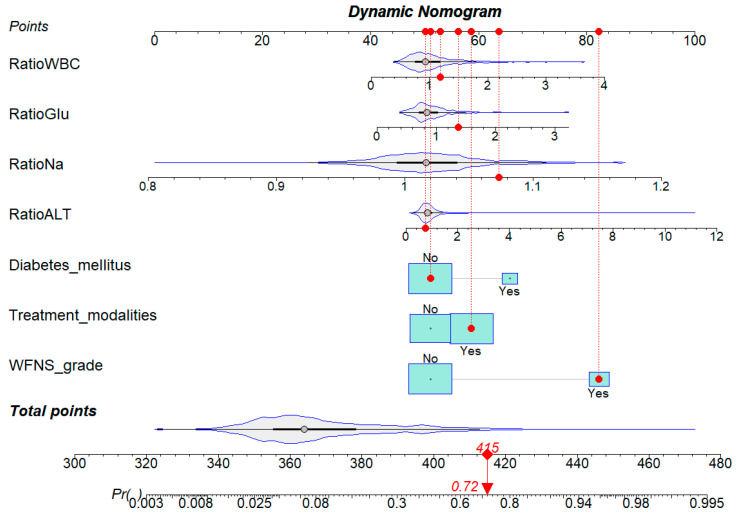
Nomogram based on Model 3.

**Figure 4:**
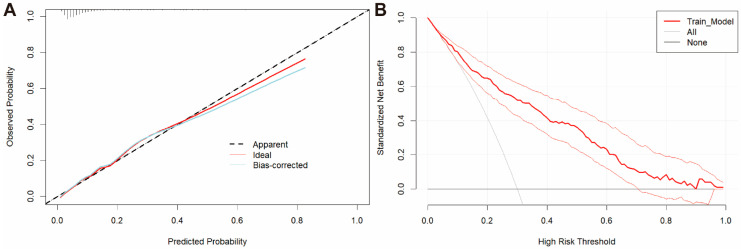
(**A**) Calibration curve of Model 3 in the derivation cohort. (**B**) DCA curve of Model 3 in the derivation cohort, with reference lines representing the net benefit of the default strategy, which is the net benefit obtained when no predictions are made. “None” represents the scenario where all patients are considered negative, and no interventions are applied. “All” represents the scenario where all patients are considered positive, and interventions are always applied.

To enable individualized risk estimation for unfavorable outcomes, nomograms were developed to visually represent the predictions of Model 3 (Figure 3). The ROC curve analysis showed that the nomogram achieved an excellent predictive performance (AUC = 0.839; 95% CI, 0.810–0.866). The Hosmer–Lemeshow test yielded a *p*-value of 0.908, indicating a good model fit. Additionally, an internal calibration plot with 1000 bootstrap iterations (Figure 4A) demonstrated strong agreement between predicted and actual unfavorable outcomes. DCA curves demonstrated substantial clinical benefits within risk thresholds from 0.1 to 0.7 (Figure 4B). Internal validation using 1000 bootstrap samples produced a mean AUC of 0.822, with validated AUCs ranging from 0.714 to 0.923, confirming the model’s excellent discriminatory ability.

**Figure 5:**
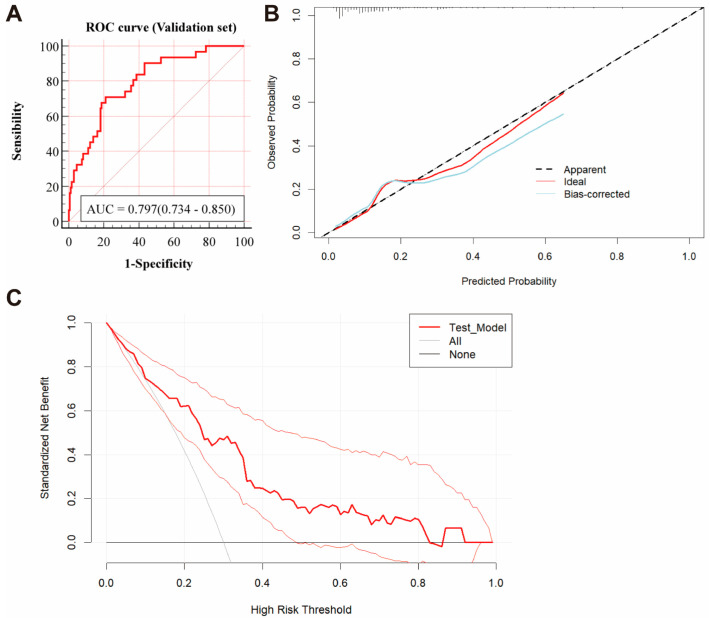
(**A**) ROC curve of Model 3 in the validation set. (**B**) Calibration curve of Model 3 in the validation set. (**C**) The DCA curve of Model 3 in the validation set, with reference lines representing the net benefit of the default strategy, which is the net benefit obtained when no predictions are made. “None” represents the scenario where all patients are considered negative, and no interventions are applied. “All” represents the scenario where all patients are considered positive, and interventions are always applied.

To further assess the efficacy and generalizability of Model 3, we conducted validations in a validation cohort. As shown in Figure 5A, the AUC was 0.797 (95% CI, 0.734–0.850) in the validation cohort. The calibration curve (Figure 5B) demonstrated good agreement between predicted and observed probabilities, while the decision curve analysis (DCA) (Figure 5C) indicated substantial clinical utility across risk thresholds from 0.2 to 0.5. These findings confirm that the nomogram model’s strong performance extends beyond the derivation cohort.

## 4. Discussion

This single-center retrospective study explored the association among clinical parameters, perioperative changes in laboratory indicators, and unfavorable 6-month outcomes in patients with aSAH. The key findings are as follows: (1) identification of perioperative changes in ALT, sodium, glucose, and WBC as independent prognostic factors for unfavorable outcomes, and (2) the development and validation of novel nomogram models that incorporate these perioperative changes, demonstrating a superior predictive performance.

### 4.1. Clinical Parameters Are Risk Factors for Unfavorable Outcomes

In our study, we identified several clinical parameters associated with poor outcomes in patients, including diabetes mellitus, WFNS grade, and treatment modality. The WFNS grade, which reflects the neurological status, is widely used to predict outcomes in patients with aSAH. A high WFNS grade is a significant predictor of poor prognosis in this patient population [17]. In addition, aSAH patients with diabetes mellitus face a significantly higher risk of developing cerebral vasospasm and delayed cerebral ischemia, conditions that are associated with a poor outcome [18,19]. The International Subarachnoid Aneurysm Trial (ISAT) evaluated 2143 patients with ruptured aneurysms, finding that endovascular coiling was more likely to achieve independent survival at 1 year compared to surgical clipping [4]. Similarly, a study by Yuanli Zhao et al. supports this conclusion in a Chinese cohort. Additionally, their research indicates that patients in the surgical clipping group experienced more in-hospital complications, a fact which may have contributed to the poorer functional prognosis observed in these patients [20]. In line with previous studies, our research also found that endovascular coiling is associated with improved functional outcomes.

### 4.2. Perioperative Changes in Laboratory Indicators Are Risk Factors for Unfavorable Outcomes

Both aSAH itself and therapeutic interventions impact patient prognosis by triggering complex pathophysiological mechanisms, including metabolic dysregulation, electrolyte imbalances, systemic inflammation, and multiorgan damage [14,21]. This highlights the importance of identifying reliable prognostic biomarkers, which could significantly enhance risk stratification and inform therapeutic decision making.

Most previous studies investigating the relationship between laboratory indicators and functional outcomes have primarily focused on admission indicators [10,22]. Although most prognostic factors are established at admission and are generally unmodifiable, therapeutic interventions play a significant role in shaping patient outcomes. These interventions, whether surgical or endovascular, are essential in managing the progression of the disease and preventing complications. The impact of these therapeutic approaches, however, may not always be immediately apparent, but can be reflected in the perioperative changes in laboratory indicators. Thus, monitoring these changes provides valuable insights into the effectiveness of the intervention and can help guide clinical decision making. [9,23,24]. Our study incorporates these factors and highlights the potential predictive value of changes in WBC count and ALT, glucose, and sodium concentrations for unfavorable outcomes. ALT, a marker of liver function, can be elevated due to systemic inflammatory responses in patients with aSAH. The mechanism by which liver injury contributes to a poor prognosis in patients with SAH remains unclear. While reduced liver function may lead to coagulation abnormalities, increasing the risk of bleeding complications, one study has suggested that liver injury following cerebral hemorrhage is relatively mild and may not significantly impact coagulation function in patients. A previous study has shown that liver function may be impaired following hemorrhagic stroke, evidenced by elevated levels of AST and alkaline phosphatase [25]. Additionally, Zheng et al. reported that elevated concentrations of ALT are related to outcome in elderly patients with aSAH [26]. Consistent with these findings, our analysis indicates that perioperative elevations in alanine aminotransferase are significantly associated with unfavorable outcomes, with an odds ratio of 1.41.

Serum sodium fluctuations are common after aSAH and have been extensively studied for their impact on patient outcomes. Fluctuations in sodium levels may impact the prognosis of aSAH patients through multiple mechanisms, including exacerbating cerebral edema and delayed cerebral ischaemia [27,28]. The prognostic implications of both hyponatremia and hypernatremia remain topics of ongoing debate [7,27]. However, fluctuations in serum sodium levels have been implicated in the development of delayed cerebral ischemia and adverse clinical outcomes in aSAH [9]. Consistent with these findings, our study demonstrates that elevated sodium levels following treatment are significantly associated with poor outcomes (OR = 5.40, *p* < 0.001).

Research has shown that glucose metabolism abnormalities, including stress-induced hyperglycemia, are common in patients with aSAH and may accelerate disease progression [29]. Stress hyperglycemia is a temporary disruption in glucose regulation triggered by acute stress, arising not only from the disease itself but also from therapeutic interventions [30]. Persistent hyperglycemia after aSAH aggravates blood–brain barrier disruption, delayed cerebral ischemia, and secondary brain injury, leading to a poor prognosis for the patient [31,32,33]. Historically, studies have primarily focused on blood glucose levels measured at admission, overlooking the effects of subsequent therapeutic interventions [8,25]. Moreover, previous research has linked elevated admission glucose levels and sustained hyperglycemia to a higher risk of unfavorable outcomes [14]. Building on these findings, our study demonstrates that postoperative increases in serum glucose concentrations are significantly associated with poorer outcomes.

Subarachnoid hemorrhage has been shown to trigger a systemic inflammatory response which is closely associated with unfavorable outcomes [34]. Additionally, therapeutic interventions, particularly surgical clipping, further stimulate an inflammatory response. The inflammatory response exacerbates neuroinflammation, contributing to neuronal injury and to the disruption of the blood–brain barrier, both of which could significantly impact the prognosis of a stroke [35]. As a key player in inflammation, white blood cells play a critical role in this process. It has been proven that elevated WBC counts at admission are significantly associated with a higher risk of poor 3-month outcomes [11]. Our findings indicate that elevated WBC counts during the perioperative period are significantly associated with unfavorable outcomes in patients with aSAH. Notably, perioperative WBC levels were identified as an independent risk factor for poor prognosis, with elevated postoperative WBC counts strongly associated with unfavorable patient outcomes.

The 2018 multinational Subarachnoid Hemorrhage International Trialists’ study developed a series of additive models that combine patient characteristics, imaging findings, and treatment factors to predict outcomes in aSAH patients, each demonstrating strong discriminatory power. However, those models did not incorporate objective laboratory tests, although laboratory indicators appear to be critical predictors in the study of aSAH [17]. To assess whether perioperative changes in laboratory indicators enhance the predictive performance of our models, we developed three models by sequentially incorporating patient functional status scores, demographic parameters, and laboratory indicators. The results demonstrate an improvement in the model’s AUC value from 0.715 to 0.839 as additional variables were incorporated, with perioperative changes in laboratory indicators contributing significantly to this enhancement. In conclusion, we propose that perioperative changes in laboratory indicators capture the impact of therapeutic interventions and are closely associated with the 6-month functional prognosis. The prognostic model developed from these indicators, combined with other clinical parameters, offers a reliable tool for predicting poor outcomes following aSAH.

### 4.3. The Nomogram Model to Predict Poor Functional Outcomes

This study developed and validated novel nomogram models to predict poor functional outcomes at 6 months in aSAH patients. The developed nomogram model, which includes diabetes mellitus, WFNS grade, treatment modality, and perioperative changes in laboratory indicators (ALT, sodium, glucose, and WBC), demonstrated a strong predictive performance, with an AUC value of 0.839 in the derivation cohort and 0.797 in the validation cohort. Calibration curves showed good concordance between predicted and observed probabilities of unfavorable outcomes. Among the computational models for outcome prediction, the nomogram is a valuable and user-friendly tool, as it graphically represents the model and offers a more precise probability calculation for clinical events compared to conventional odds ratios (ORs). In terms of clinical utility, the nomogram’s ability to swiftly calculate the probability of a clinical event through a simple linear scoring system—unlike machine learning models that often require complex software and computational tools—makes it highly suitable for rapid application in clinical settings [16,36]. In summary, by inputting patient-specific data into the model, clinicians can obtain a total score which can be translated into the probability that a patient will have a poor functional prognosis within six months, enabling a more accurate risk assessment for individual patients.

### 4.4. Limitations

This study has several limitations. Firstly, as a retrospective, single-center study, it may be subject to selection bias, and the non-randomized intervention limits causal inference. Secondly, the findings may not be generalizable beyond the Chinese population. Although Model 3 shows some extrapolation potential through internal validation, the lack of external validation and the focus on a Chinese population limit its generalizability. Thirdly, despite our efforts to expand the scope of laboratory data records, due to limited clinical data, certain laboratory parameters, such as hyperoxia, CRP–albumin ratio, temperature, inflammatory markers, and heart rate variability, were not assessed. Furthermore, our analysis evaluated outcomes only at 6 months, without addressing potential long-term progression. Future prospective multicenter studies are needed to validate these findings.

## 5. Conclusions

In this study, diabetes mellitus, WFNS grade, treatment modality, and perioperative changes in laboratory indicators (ALT, sodium, glucose, and WBC) are identified as independent predictors of unfavorable outcomes in aSAH patients, enhancing the predictive ability of existing clinical prognostic models. Additionally, the nomogram models developed for predicting 6-month poor functional outcomes demonstrate strong discrimination, accuracy, and clinical utility, aiding physicians in identifying high-risk patients, guiding treatment decisions, and suggesting potential directions for future studies. In the future, an international, multicenter validation of the nomogram model in a larger study population will be essential to provide robust evidence supporting its clinical application.

## Figures and Tables

**Table 1:** Univariate analysis of baseline characteristics in the derivation cohort stratified by the 6-month functional outcomes.

Variables	Good Functional Outcome (*n* = 603)	Poor Functional Outcome (*n* = 101)	*p*
Age (years)	55.05 ± 11.64	58.11 ± 11.99	0.015
Female sex	355 (58.87)	67 (66.34)	0.157
Smoking	148 (24.54)	24 (23.76)	0.866
Alcohol	116 (19.24)	17 (16.83)	0.568
Hypertention	341 (56.55)	68 (67.33)	0.042
Hyperlipidemia	57 (9.45)	11 (10.89)	0.651
Diabetes mellitus	63 (10.45)	18 (17.82)	0.032
WFNS grade 3–5	69 (11.44)	55 (54.46)	<0.001
Location			0.527
Anterior cerebral artery	199 (33.00)	26 (25.74)	
Internal carotid artery	201 (33.33)	38 (37.62)	
Middle cerebral artery	104 (17.25)	20 (19.80)	
Posterior circulation	99 (16.42)	17 (16.83)	
Modified Fisher grade 3–4	456 (75.62)	101 (100.00)	<0.001
Treatment modalities			0.008
Coiling	295 (48.92)	35 (34.65)	
Clipping	308 (51.08)	66 (65.35)	

**Table 2:** Univariate analysis of perioperative laboratory indicator changes in the derivation cohort stratified by the 6-month functional outcomes.

Variables	Good Functional Outcome (n = 603)	Poor Functional Outcome (n = 101)	*p*
D-dimer	1.92 (1.14, 3.21)	1.79 (1.04, 3.57)	0.818
FDP	1.12 (0.98, 1.34)	1.20 (1.05, 1.42)	0.010
ALT	0.81 (0.71, 0.97)	0.90 (0.75, 1.16)	0.002
AST	0.83 (0.68, 1.11)	1.01 (0.72, 1.26)	0.009
Sodium	1.01 (0.99, 1.04)	1.03 (1.02, 1.08)	<0.001
Potassium	0.96 (0.89, 1.05)	1.00 (0.93, 1.07)	0.016
eGFR	1.02 (0.97, 1.06)	0.99 (0.94, 1.03)	<0.001
Glucose	0.83 (0.71, 1.01)	0.91 (0.71, 1.14)	0.091
WBC	0.93 (0.74, 1.15)	1.00 (0.84, 1.24)	0.011
NLR	0.76 (0.47, 1.35)	0.98 (0.58, 1.66)	0.012
RBC	0.93 (0.88, 0.99)	0.93 (0.87, 0.99)	0.539
HGB	0.93 (0.88, 0.99)	0.92 (0.86, 0.99)	0.537
PLT	0.92 (0.82, 1.01)	0.90 (0.79, 1.01)	0.241

**Table 3:** Multivariate logistic regression analysis for 6-month poor functional outcomes.

Variables	β	OR (95% CI)	*p*
Diabetes mellitus	1.04	2.84 (1.44–5.59)	0.002
WFNS grade 3–5	2.22	9.17 (5.49–15.33)	<0.001
Treatment modalities			
Coiling		1.00 (Reference)	
Clipping	0.54	1.71 (1.03–2.85)	0.037
ALT	0.34	1.41 (1.04–1.91)	0.028
Sodium ^†^	16.87	5.40 (3.01–9.71)	<0.001
Glucose	0.78	2.18 (1.05–4.53)	0.037
WBC	0.77	2.15 (1.17–3.96)	0.014

^†^ To enhance interpretability, we adjusted the OR to reflect a 0.1 unit change. This adjustment was necessary because the range of variation for sodium was narrow, resulting in a particularly high OR value. Formulas: OR = exp(β × 0.1); 95% CI = exp(0.1 × [β ± 1.96 × SE]).

## Data Availability

The datasets used and/or analyzed during the current study are available from the corresponding author upon reasonable request.

## Supplementary Materials

The following supporting information can be downloaded at: https://www.mdpi.com/article/10.3390/jcm14051443/s1. Table S1: Baseline comparison between the training set and the validation set.
