# Supplementary material for: Development of a Clinical and Laboratory-Based Predictive Nomogram Model for Unfavorable Functional Outcomes Among Patients Who Undergo Interventions for Aneurysmal Subarachnoid Hemorrhage
Source: J Clin Med. 2025 Feb 21;14(5):1443. doi: 10.3390/jcm14051443 (PMC11900520; doi:10.3390/jcm14051443)
Supplement: Supplementary file 1 [file jcm-14-01443-s001.zip › jcm-3402304-supplementary.pdf]

Table S1 Baseline comparison between training set and validated set

Variables	Training set (n = 704)	Validated set(n = 302)	*P*
Group			0.245
Good functional outcome	603 (85.65)	250 (82.78)	
Poor functional outcome	101 (14.35)	52 (17.22)	
Age	56.25 ± 11.21	55.49 ± 11.73	0.342
Female sex	422 (59.94)	177 (58.61)	0.693
Smoking	172 (24.43)	81 (26.82)	0.423
Alcohoal	133 (18.82)	72 (23.84)	0.074
Hypertention	409 (58.09)	163 (53.97)	0.226
Hyperlipidemia	68 (9.66)	24 (7.95)	0.388
Diabetes mellitus	81 (11.51)	26 (8.61)	0.172
WFNS grade 3-5	124 (17.61)	57 (18.87)	0.633
Location, n(%)			0.965
Anterior cerebral artery	225 (31.96)	101 (33.44)	
Internal carotid artery	239 (33.95)	102 (33.78)	
Middle cerebral artery	124 (17.61)	52 (17.22)	
Posterior circulation	116 (16.48)	47 (15.56)	
Modified Fisher grade 3–4	557 (79.12)	242 (80.13)	0.716
Treatment modalities			0.805
Coiling	330 (46.88)	139 (46.03)	
Clipping	374 (53.13)	163 (53.97)	
Laboratory tests			
D-dimer	1.89 (1.14, 3.29)	2.02 (1.12, 3.13)	0.858
FDP	1.12 (0.99, 1.35)	1.11 (0.97, 1.34)	0.415
ALT	0.82 (0.71, 1.00)	0.84 (0.71, 0.98)	0.814
AST	0.84 (0.69, 1.16)	0.86 (0.69, 1.13)	0.820
Sodium	1.02 (0.99, 1.04)	1.01 (0.99, 1.03)	0.142
Potassium	0.97 (0.89, 1.06)	0.95 (0.89, 1.04)	0.271
eGFR	1.01 (0.97, 1.06)	1.01 (0.96, 1.06)	0.556
Glucose	0.84 (0.71, 1.03)	0.85 (0.71, 0.99)	0.864
WBC	0.93 (0.76, 1.17)	0.92 (0.77, 1.21)	0.625
NLR	0.79 (0.49, 1.40)	0.79 (0.47, 1.38)	0.680
RBC	0.93 (0.88, 0.99)	0.94 (0.87, 0.99)	0.783
HGB	0.93 (0.87, 0.99)	0.94 (0.87, 0.99)	0.995
PLT	0.92 (0.82, 1.01)	0.92 (0.82, 1.01)	0.983
